# Evaluation of the Existing Electrophysiological Severity Classifications in Carpal Tunnel Syndrome

**DOI:** 10.3390/jcm11061685

**Published:** 2022-03-18

**Authors:** Toru Sasaki, Takafumi Koyama, Tomoyuki Kuroiwa, Akimoto Nimura, Atsushi Okawa, Yoshiaki Wakabayashi, Koji Fujita

**Affiliations:** 1 Department of Orthopaedic and Spinal Surgery, Graduate School of Medical and Dental Sciences, Tokyo Medical and Dental University, 1-5-45, Yushima, Bunkyo-ku, Tokyo 113-8519, Japan; t-sasaki.orth@tmd.ac.jp (T.S.); koya.orth@tmd.ac.jp (T.K.); kurorth@gmail.com (T.K.); okawa.orth@tmd.ac.jp (A.O.); 2 Department of Orthopaedic Surgery, Tsuchiura Kyodo General Hospital, 4-1-1, Tsuchiura 300-0028, Ibaraki, Japan; 3 Department of Functional Joint Anatomy, Graduate School of Medical and Dental Sciences, Tokyo Medical and Dental University, 1-5-45, Yushima, Bunkyo-ku, Tokyo 113-8519, Japan; nimura.orj@tmd.ac.jp; 4 Department of Orthopaedic Surgery, Yokohama City Minato Red Cross Hospital, 3-12-1, Shinyamashita, Naka-ku, Yokohama City 231-8682, Kanagawa, Japan; dr_waka@cl.cilas.net

**Keywords:** carpal tunnel syndrome, nerve conduction study, median nerve, electrophysiological severity classification

## Abstract

Electrophysiological examination is important for the diagnosis and evaluation of nerve function in carpal tunnel syndrome (CTS). Electrophysiological severity classifications of CTS using a nerve conduction study (NCS) have been reported, and there are many reports on the relationship between severity classifications and clinical symptoms. The existing electrophysiological severity classifications have several problems, such as cases that do not fit into a classification and unclear reasons for the boundary value. The purpose of this study was to clarify the relationship between sensory nerve conduction velocity (SCV) and distal motor latency (DML) and to evaluate whether the existing severity classification method is appropriate. We created a scatter diagram between SCV and DML for our NCSs and found a negative correlation between SCV and DML (correlation coefficient, −0.786). When we applied our NCSs to the existing classifications (Padua and Bland classifications), there were many unclassifiable cases (15.2%; Padua classification), and the number of Grade 3 cases was significantly higher than that of Grade 2 or 4 cases (Bland classification). Our large dataset revealed a strong negative correlation between SCV and DML, indicating that the existing severity classifications do not always accurately reflect the severity of the disease.

## 1. Introduction

Carpal tunnel syndrome (CTS) is identified by numbness, pain, and disordered thumb opposition associated with localized compression of the median nerve at the wrist and is the most common nerve entrapment syndrome [1,2,3,4,5,6]. Although CTS is diagnosed mainly based on clinical findings, an electrophysiological examination is important for diagnosis and determining the appropriate course of treatment [7,8,9,10]. Electrophysiological severity classification of CTS using a nerve conduction study (NCS) is a useful method that can easily display results of electrophysiological examination with several parameters on a single scale, and various classification methods have been reported [6,11,12,13]. In addition, there have been many reports on the assessment of clinical symptoms using electrophysiological severity classifications [14,15,16,17].

A previous study reported that because sensory fibers have a large proportion of large myelinated fibers, they are more susceptible to ischemic damage [6,18]. In clinical practice, numbness due to sensory disturbance is more likely to be recognized at an early stage than muscle atrophy due to motor disturbance [19,20]. Therefore, many existing severity classifications are based on the premise that CTS is a disorder of sensory fiber dominance [6,11,12,13]. However, motor nerve fiber damage appears long before the appearance of muscle atrophy, and sensory nerve damage does not necessarily occur first. The reasons for the boundary value that separates severities are not clear in the existing classifications, and we have had cases in which patient results do not fit the existing classifications. We thought it necessary to examine whether or not the existing severity classifications can accurately evaluate the degree of disability in CTS patients.

We hypothesized that the existing electrophysiological severity classifications have several problems such as the existence of cases that do not fit the classifications and unclear reasons for the boundary value. The purpose of this study was to determine the relationship between sensory nerve conduction velocity (SCV) and distal motor latency (DML) using data from NCSs on patients with CTS at our hospital and apply the NCS data to the existing severity classifications (Padua and Bland classifications) to evaluate whether the existing classifications are appropriate classification methods.

## 2. Materials and Methods

### 2.1. Participants

In this retrospective study, all clinical and NCS data were obtained from the 560 patients (1120 hands; median age, 69.5 years; 264 male hands, 856 female hands) treated at the department of orthopedic surgery at Tokyo Medical and Dental University (Table 1). We obtained written informed consent from all the participants. This retrospective analysis was approved by our institutional review board. This study included patients who were suspected of having CTS by hand surgeons and underwent NCSs between April 2007 and September 2019 at our hospital. The inclusion criteria for this study were as follows: clinical symptoms of CTS (numbness, tingling, and pain) and positive examination findings for CTS, including a positive Phalen test and Tinel’s sign. In addition to the patients who underwent NCSs preoperatively, the patients who had CTS symptoms, underwent NCSs postoperatively, and had abnormal results were also included in this study. The patients whose symptoms disappeared after surgery and had normal SCV and DL were excluded. Since the purpose of the study was to electrophysiologically evaluate sensory and motor neuropathies at the carpal tunnel area, patients with peripheral polyneuropathy, cervical disease, de Quervain syndrome, or trigger finger and those with a history of a distal radial fracture were also included in this study. Although peripheral polyneuropathy and cervical disease may cause abnormal results in NCSs, we included them in our analysis. In addition, although patients with de Quervain syndrome or trigger finger and those with a history of a distal radial fracture may have wrist pain, numbness, and a positive Tinel’s sign at the carpal tunnel, we included these patients. This is because the participants of this study were diagnosed by hand surgeons as having neuropathy in the carpal tunnel area, and the main neuropathy is considered to be entrapment of the median nerve in the carpal tunnel area even if there are other comorbidities. Patients were excluded from this study if they could not undergo NCSs due to pain caused by electrical stimulation.

### 2.2. Nerve Conduction Study

NCSs were performed on both hands of each patient by trained clinical technicians, with patients relaxed and in the supine position. A skin temperature of 32 °C was maintained on the dorsum of the hand. An NCS of both median nerves was performed using an evoked potential/electromyography system (MEB-2300; Nihon Kohden, Tokyo, Japan) with the bandpass filter set to 5–10 Hz for motor nerve recording and 2–20 Hz for sensory recording. The sensory nerve action potential of the median nerve was antidromically recorded with a pair of cup electrodes placed on the index finger. Square-pulse supramaximal electrical stimuli at 0.5 Hz with a duration of 0.3 ms were delivered to the palm, wrist, and elbow. We calculated the sensory nerve conduction velocity (SCV) from the latency of the waveform of the sensory nerve action potential and the distance between the stimulation point and the recording electrode. The compound muscle action potential was recorded with a pair of surface cup electrodes placed over the abductor pollicis brevis by using the belly–tendon method. Square-pulse supramaximal electrical stimuli at 0.5 Hz with a duration of 0.3 ms were delivered to the wrist and elbow. The wrist stimulation point was 7 cm proximal to the cathode electrode placed on the abductor pollicis brevis. Distal motor latency (DML) was determined from the waveform of the compound motor action potential. Each measurement was performed twice to confirm reproducibility. The neurologists and orthopedic surgeons calculated the SCV and DML values from the waveforms. Those with unclear latency and no reproducibility were considered non-measurable. 

### 2.3. Analysis

First, we analyzed the relationship between SCV and DML from the 1120 right and left hands of 560 patients. The results of the NCSs on the healthy side were also included in the analysis. We created a scatter diagram to study the correlation between SCV and DML using data from 934 hands in which both SCV and DML were measurable. The correlation coefficient of this scatter diagram was calculated, and the relationship between SCV and DML was evaluated. Furthermore, only patients with diabetes were extracted, and a scatter diagram was created in the same way. We added an analysis that excluded patients with peripheral polyneuropathy or cervical disease and postoperative patients. We also created a scatter diagram for these patients.

**Figure 1:**
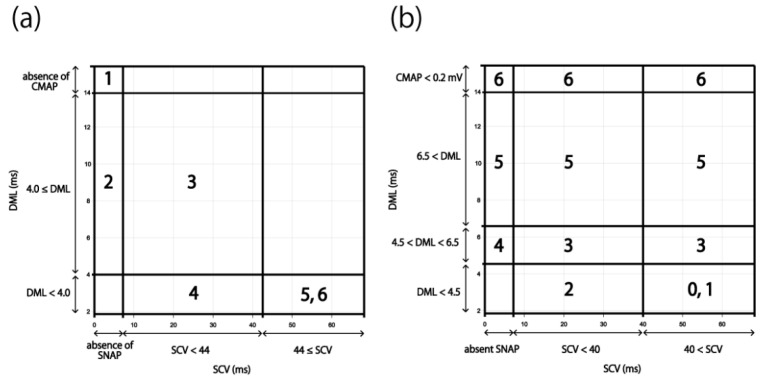
Electrophysiological severity classifications for CTS. (**a**) Padua classification. 1. Extreme CTS: absence of SNAP and CMAP. 2. Severe CTS: absence of SNAP and DML ≥ 4.0 ms. 3. Moderate CTS: SCV < 44 m/s and DML ≥ 4.0 ms. 4. Mild CTS: SCV < 44 m/s and DML < 4.0 ms. 5. Minimal CTS: “standard negative” hands with abnormal comparative or segmental tests. 6. Negative: normal findings on all tests. (**b**) Bland classification. Grade 0: no neurophysiological abnormalities. Grade 1: very mild CTS, detected only in two sensitivity tests (e.g., inching, palm/wrist median/ulnar comparison, ring finger “double peak”). Grade 2: mild CTS (SCV < 40 m/s and DML < 4.5 ms). Grade 3: moderately severe CTS (4.5 ms < DML < 6.5 ms and SCV < 40 m/s). Grade 4: severe CTS (4.5 ms < DML < 6.5 ms and absent SNAP). Grade 5: very severe CTS (6.5 ms < DML). Grade 6: extremely severe CTS (CMAP < 0.2 mV). Abbreviations: CMAP, compound muscle action potential; CTS, carpal tunnel syndrome; DML, distal motor latency; SCV, sensory nerve conduction velocity; SNAP, sensory nerve action potential.

The NCS results of 1120 hands were then applied to the existing severity classifications (Padua and Bland classifications). A six-level classification (Grades 1–6) was created in the Padua classification. The boundary values of SCV and DML were set to 44 m/s and 4.0 ms, respectively [12] (Figure 1a). In the Bland classification, a seven-level classification (Grades 0–6) was created. The boundary values of SCV and DML were set to 40 m/s and 4.5–6.5 ms, respectively [11] (Figure 1b). We counted the number of cases that could not fit these classifications and extracted the problems of these systems by applying the results of our NCSs to the two classifications.

## 3. Results

NCSs were performed on 1120 hands. The participants’ demographic characteristics are presented in Table 1. There were 28 patients with diabetes (56 hands), 42 patients with cervical desease (84 hands), 60 patients with de Quervain syndrome or trigger finger (120 hands), and nine patients with a history of a distal radial fracture (18 hands). There were 247 preoperative patients (494 hands) and 313 postoperative patients (626 hands).

### 3.1. Relationship between SCV and DML

**Figure 2:**
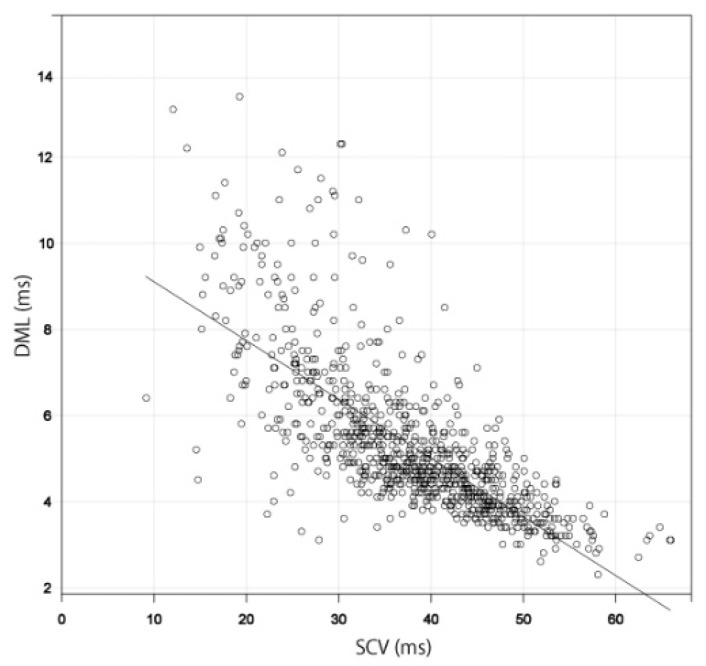
Scatter diagram between SCV and DML. The straight line shows the correlation between SCV and DML. Abbreviations: DML, distal motor latency; SCV, sensory nerve conduction velocity.

**Figure 3:**
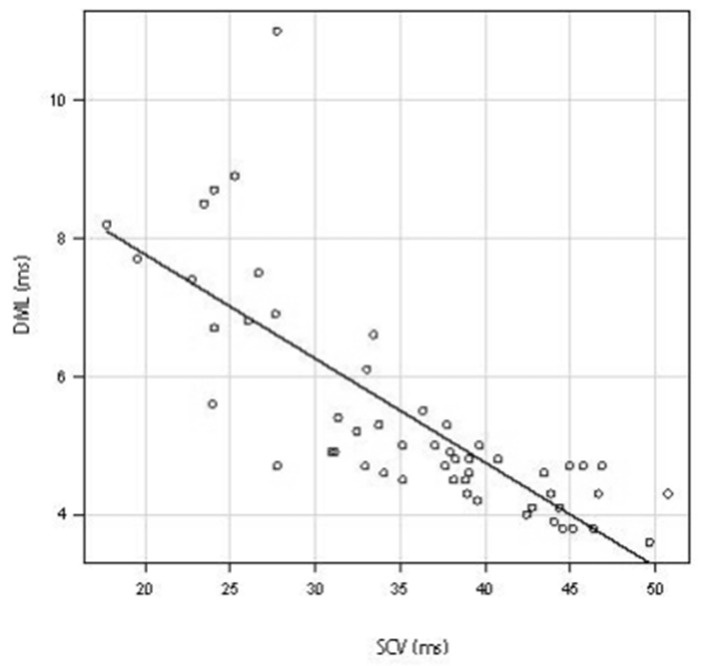
Scatter diagram between SCV and DML in the diabetes patients. The straight line shows the correlation between SCV and DML. Abbreviations: DML, distal motor latency; SCV, sensory nerve conduction velocity.

**Figure 4:**
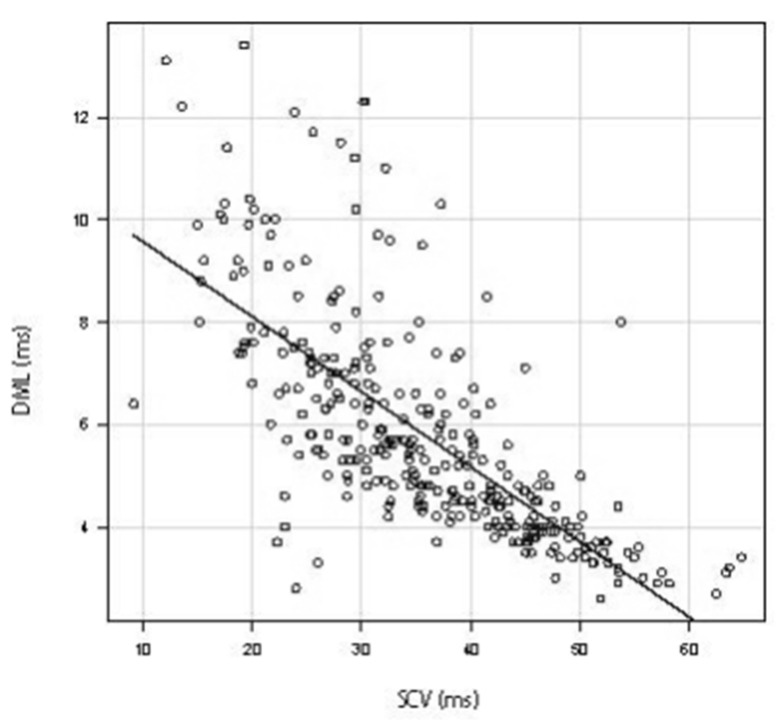
Scatter diagram between SCV and DML that excluded the patients with peripheral polyneuropathy or cervical disease and the postoperative patients. The straight line shows the correlation between SCV and DML. Abbreviations: DML, distal motor latency; SCV, sensory nerve conduction velocity.

A scatter diagram was created using the results for 934 hands in which both SCV and DML were measurable. Both the SCV and DML values showed a strong negative correlation with a Spearman’s rank correlation coefficient of −0.786 (Figure 2) [21,22]. The scatter diagram of diabetes patients also showed a strong negative correlation between SCV and DML (53 hands) (Spearman’s rank correlation coefficient of −0.841) (Figure 3). The scatter diagram that excluded the patients with peripheral polyneuropathy or cervical disease and the postpoerative patients also showed a strong negative correlation between SCV and DML (320 hands) (Spearman’s rank correlation coefficient of −0.779) (Figure 4).

### 3.2. Applying the Results to the Existing Severity Classifications 

**Figure 5:**
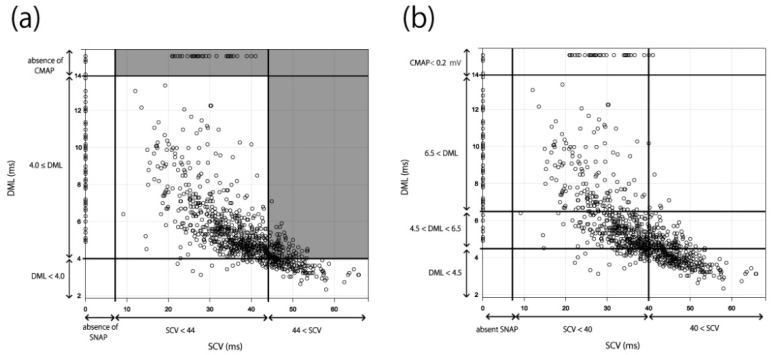
Results applied to the existing classifications. The NCS results of 1120 cases were applied to the existing severity classifications (Figure 1). (**a**) Padua classification: gray areas indicate cases that could not be classified. (**b**) Bland classification. Abbreviations: CMAP, compound muscle action potential; DML, distal motor latency; NCS, nerve conduction study; SCV, sensory nerve conduction velocity; SNAP, sensory nerve action potential.

In the Padua classification, cases with normal SCV but abnormal DML and cases with measurable SCV but non-measurable DML were unclassifiable; thus, 170 of the 1120 cases (15.2%) could not be classified (Figure 5a). In the Bland classification, all the cases could be classified (Figure 5b), including 260 cases that were Grade 0 or 1, 59 cases that were Grade 2, 449 cases that were Grade 3, 10 cases that were Grade 4, 248 cases that were Grade 5, and 94 cases that were Grade 6. The number of Grade 3 cases was significantly higher than that of Grade 2 or 4 cases.

## 4. Discussion

In this study, we showed that SCV and DML had a strong negative correlation using the NCS results of the CTS patients (all patients, diabetic patients, andpatients who were excluded postoperatively) and those with peripheral polyneuropathy or cervical disease. We applied the results to the existing severity classification, and the following problems were identified: unclassifiable cases in the Padua classification and bias of the number in the Bland classification.

A previous study reported that because sensory fibers had a large proportion of large myelinated fibers, they were more susceptible to ischemic damage [6,18]; the Padua and Bland classifications were based on this premise [11,13]. However, our results show that SCV and DML have a strong negative correlation, indicating that we could not conclude whether the sensory fibers or motor fibers are damaged first. When our NCS results were applied to the existing severity classifications, 15.2% of cases could not be categorized using the Padua classification. Cases with normal SCV but abnormal DML and cases with measurable SCV but non-measurable DML were unclassifiable. The high number of unclassifiable cases was due to the fact that the Padua classification was based on sensory fiber dominance. Similarly, the Stevens and Werner classifications, which are cited in many papers, are based on the premise that sensory nerves are damaged first, and unclassifiable cases are also observed in these classifications [6,13]. According to previous reports, Martin–Gruber anastomosis (MGA) is present in 5–40% of patients, which may lead to prolonged DML and normal or higher SCV values [23]. Given the influence of MGA and our results suggesting that SCV and DML are strongly correlated, it may not be appropriate to use these classifications in clinical assessment. 

**Figure 6:**
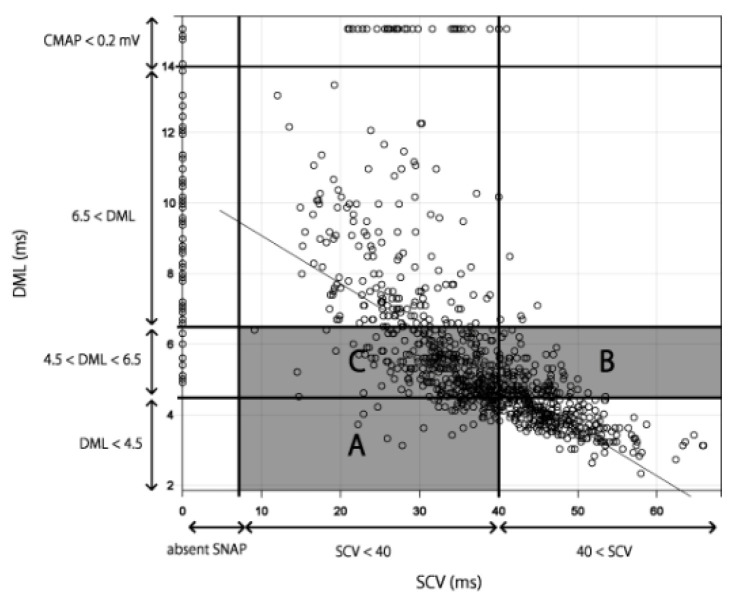
Analysis of the Bland classification. Area A: Grade 2 in Figure 1b. Area B: normal SCV in Grade 3 in Figure 1b. Area C: SCV < 40 m/s in Grade 3 in Figure 1b. The straight line shows the correlation between SCV and DML in Figure 2. Abbreviations: CMAP, compound muscle action potential; DML, distal motor latency; SCV, sensory nerve conduction velocity; SNAP, sensory nerve action potential.

Although all the results of NCSs were categorized in the Bland classification, as Bland himself states, a motor terminal latency measurement of 6.5 ms was arbitrary and the reasons for the boundary values (DML: 4.5, 6.5 ms, SCV: 4.0 m/s) were not clearly reported [11]. Furthermore, the Bland classification does not reflect the severity of disease in the order of classification. In the Bland classification, the cases in which sensory fibers were damaged first were classified as Grade 2 (area A of Figure 6), and those in which motor fibers were damaged first were classified as Grade 3 (area B of Figure 6). In other words, the cases in which motor fibers were damaged first were considered more severe in the Bland classification. However, motor and sensory fibers had a strong correlation in our results; thus, cases in area B were not necessarily more severe than those in area A (Figure 6). Furthermore, in Figure 6, the cases in areas B and C were classified as Grade 3 using the Bland classification. However, it is not necessarily correct to state that cases in areas B and C have the same severity because most cases in area C were more severe than those in area B when taking the correlation line into consideration. Because areas B and C were classified as having the same severity (Grade 3), the number of Grade 3 cases may have been notably higher than that of Grades 2 and 4 when using the Bland classification. Although Bland allows individual laboratories to define abnormal sensory and motor conduction values for Grades 2, 3, and 4, adjusting the definition for abnormal values will not solve this problem.

An electrophysiological study provides useful information for an objective and quantitative assessment of the neurophysiological severity of CTS [24,25,26]. Median nerve conduction studies in CTS cases evaluate both sensory and motor nerve fibers. Electrophysiological severity classification in CTS is a useful method to show these evaluation parameters on a single scale [11,12], and there are many reports on clinical evaluations using severity classifications [27,28,29,30,31,32,33,34]. However, as shown in this paper, the existing severity classifications do not always accurately reflect the severity of disease, and our results suggest the need to reevaluate previous studies that used these classifications.

There was a limitation in this study: we did not consider MGA in this study. As MGA can affect the results of NCS, patients with and without MGA should be analyzed separately for an accurate evaluation of the relationship between SCV and DML. 

In conclusion, our large dataset revealed a strong negative correlation between SCV and DML in our NCSs in patients with CTS. Problems with the existing severity classifications were higlighted, such as unclassifiable cases, unclear boundary values, different classifications for the same degree of severity, and the same classifications for different degrees of severity. In the future, it will be necessary to develop a comprehensive CTS severity classification that includes physical findings and subjective symptoms, while taking into account the correlation between sensory and motor fiber disorders.

## Figures and Tables

**Table 1:** Characteristics of the participants.

	*n* = 1120 Hands
Age ^1^	69.5 (60–77)
Sex	
Male	132 patients, 264 hands
Female	428 patients, 856 hands
Pre-operation	247 patients, 494 hands
Post-operation	313 patients, 626 hands
Measurable DML and measurable SCV	934 hands
Non-measurable DML and non-measurable SCV	62 hands

^1^ Data are presented as the medians (interquartile range). Abbreviations: DML, distal motor latency; SCV, sensory nerve velocity.

## Data Availability

The data generated in this study are available from the corresponding author on reasonable request.
